# Effectiveness of Wearable Trackers on Physical Activity in Healthy Adults: Systematic Review and Meta-Analysis of Randomized Controlled Trials

**DOI:** 10.2196/15576

**Published:** 2020-07-22

**Authors:** Matilda Swee Sun Tang, Katherine Moore, Andrew McGavigan, Robyn A Clark, Anand N Ganesan

**Affiliations:** 1 College of Medicine and Public Health Flinders University Adelaide Australia; 2 Department of Cardiovascular Medicine Flinders Medical Centre Adelaide Australia; 3 College of Nursing and Health Sciences Flinders University Adelaide Australia; 4 South Australian Health and Medical Research Institute Adelaide Australia

**Keywords:** wearable activity tracker, physical activity, healthy adults, randomized controlled trials

## Abstract

**Background:**

Wearable trackers are an increasingly popular tool among healthy adults and are used to facilitate self-monitoring of physical activity.

**Objective:**

We aimed to systematically review the effectiveness of wearable trackers for improving physical activity and weight reduction among healthy adults.

**Methods:**

This review used the PRISMA (Preferred Reporting Items for Systematic Reviews and Meta-Analyses) methodology and reporting criteria. English-language randomized controlled trials with more than 20 participants from MEDLINE, CINAHL, Cochrane Library, Web of Science, PubMed, and Scopus (2000-2017) were identified. Studies were eligible for inclusion if they reported an intervention group using wearable trackers, reporting steps per day, total moderate-to-vigorous physical activity, activity, physical activity, energy expenditure, and weight reduction.

**Results:**

Twelve eligible studies with a total of 1693 participants met the inclusion criteria. The weighted average age was 40.7 years (95% CI 31.1-50.3), with 64.4% women. The mean intervention duration was 21.4 weeks (95% CI 6.1-36.7). The usage of wearable trackers was associated with increased physical activity (standardized mean difference 0.449, 95% CI 0.10-0.80; *P*=.01). In the subgroup analyses, however, wearable trackers demonstrated no clear benefit for physical activity or weight reduction.

**Conclusions:**

These data suggest that the use of wearable trackers in healthy adults may be associated with modest short-term increases in physical activity. Further data are required to determine if a sustained benefit is associated with wearable tracker usage.

## Introduction

Wearable activity trackers have rapidly emerged in the past decade as consumer devices to support self-monitoring of physical activity [1,2]. The use of these devices has increased exponentially, and the global sales of wearables in health care are expected to reach US $4.4 billion in 2019 and US $4.5 billion by 2020 [3].

In the past, structured lifestyle interventions have utilized education with behavior change techniques, provision of written information materials, and telephone counseling in a series of combination and permutation [4,5]. These interventions are successful in the short term but not in the long term, and they tend to be labor-intensive and costly [2,6,7]. Today, the availability and accessibility of wearable trackers equip consumers with the ability to monitor their physical activity along with online applications with motivational and tracking tools. Several systematic reviews have shown that wearable trackers are effective [4,6,8].

Contemporary wearable trackers differ from conventional pedometers as they are sophisticated devices providing real-time multidimensional feedback on physiological and health parameters including steps, calories burned, distance covered, active time, sleep assessment, and heart rate, and may include mobile connectivity or an internet application to provide personalized feedback reports [9].

To date, most of the literature on wearable trackers has focused on their feasibility, validity, and reliability [10] with limited data on the impact using these devices has on improving physical activity [6,11]. The primary purpose of this study is to evaluate the effectiveness of wearable trackers and their impact on physical activity levels in healthy adult populations with secondary outcomes of weight change in overweight populations.

## Methods

### Protocol and Registration

The protocol for this study is registered under PROSPERO with registration number CRD42019131868.

### Eligibility Criteria, Information Sources, Search and Study Selection

PRISMA (Preferred Reporting Items for Systematic Reviews and Meta-Analyses) [12] methods and reporting were used to perform a systematic literature search with a professional medical librarian (Multimedia Appendix 1) on English-language randomized controlled trials published between January 1, 2000, and August 1, 2017.

We considered English-language studies eligible for inclusion if they reported an intervention with at least one of the groups using wearable trackers to provide objective feedback on physical activity to the wearer, alone or in combination with other interventions to enhance physical activity. Only randomized controlled trials with more than 20 participants in the adult outpatient and community setting that reported a change in physical activity behavior (total steps, total activity, the proportion of participants at activity goal) were included. We are looking at the healthy population and therefore excluded studies that required participants to be hospitalized or confined to a research center, studies in disease populations, and obese populations. We excluded studies that were predominantly pedometer-based interventions since we were only considering the effect of wearable trackers.

### Data Collection Process and Data Items

Two authors independently abstracted three categories of variables from each of the included studies, with differences resolved by consensus: intervention variables (intervention duration); participant variables; quality variables (method of blinding control participants to step counts, the use of validity- and reliability-tested wearable trackers, the extent of affordability of wearable trackers and the extent to which co-interventions may have affected physical activity). If the study reported results from a different period, we used the final immediate post-intervention data in our primary analysis. For studies that reported different intensities of physical activity instead of steps per day, we chose the walking intensity results for the primary analysis.

### Risk of Bias and Quality Assessment

The risk of bias was assessed using the Cochrane risk of bias tool [13] across seven domains. Each domain was scored with low (L), unclear (U), or high (H) risk of bias. The domains assessed were as follows:

- Random sequence generation: Was there selective bias (biased allocation to interventions) due to inadequate generation of a randomized sequence?
- Allocation concealment: Was there selective bias (biased allocation to interventions) due to inadequate concealment of allocations prior to assignment?
- Selective outcome reporting: Was there reporting bias due to selective outcome reporting?
- Blinding outcome assessment: Was there detection bias due to knowledge of the allocated interventions by outcome assessors?
- Blinding participants and personnel: Was there performance bias due to knowledge of the allocated interventions by participants and personnel during the study?
- Incomplete outcome data: Was there attribution bias due to knowledge of the allocated interventions by outcome assessors?
- Other sources of bias: Was there bias due to problems not covered elsewhere?

The GRADE (Grades of Recommendation, Assessment, Development, and Evaluation) system was used to rank the quality of evidence for each study [14]. The GRADE approach uses five considerations (study limitations, consistency of effect, imprecision, indirectness, and publication bias). The criteria for the grade of evidence are as follows:

- High: We are very confident that the true effect lies close to that of the estimated effect.
- Moderate: We are moderately confident in the effect estimate; the true effect is likely close to the estimated effect, but there is a possibility that it is substantially different
- Low: Our confidence in the effect estimate is limited; the true effect may be substantially different from the estimated effect.
- Very low quality: we have very little confidence in the effect estimate; the true effect is likely to be substantially different from the estimate of the effect.

### Synthesis of Results

Statistical analysis was performed with Comprehensive Meta-Analysis Version 3 [15]. For each of the included studies, we calculated the effect sizes of physical activity for our primary interest outcome using steps/day, total moderate-vigorous physical activity (MVPA), activity units or physical activity energy expenditure, depending on the available results from the studies with steps/day taking priority. We calculated the summary outcomes or the standardized mean difference (SMD) (95% CI) using random-effects calculations. SMD is used since the included studies all assess the same outcome, physical activity, but they measure it in a variety of ways (steps/day, MVPA, energy expenditure). Hence, differences in means that are the same proportion of the standard deviation will have the same SMD, regardless of the actual scales used to make the measurements [16].

The I^2^ statistic was used as a measure of variability in observed effects estimates attributable to between-study heterogeneity [17]. For variables exhibiting mild heterogeneity (I^2^≤25%), pooled estimates were derived with fixed effects models. For variables exhibiting more than moderate heterogeneity (I^2^>25%), pooled estimates were derived with random-effects models. Sub-group analysis was used to assess secondary outcomes.

## Results

### Study Selection

**Figure 1:**
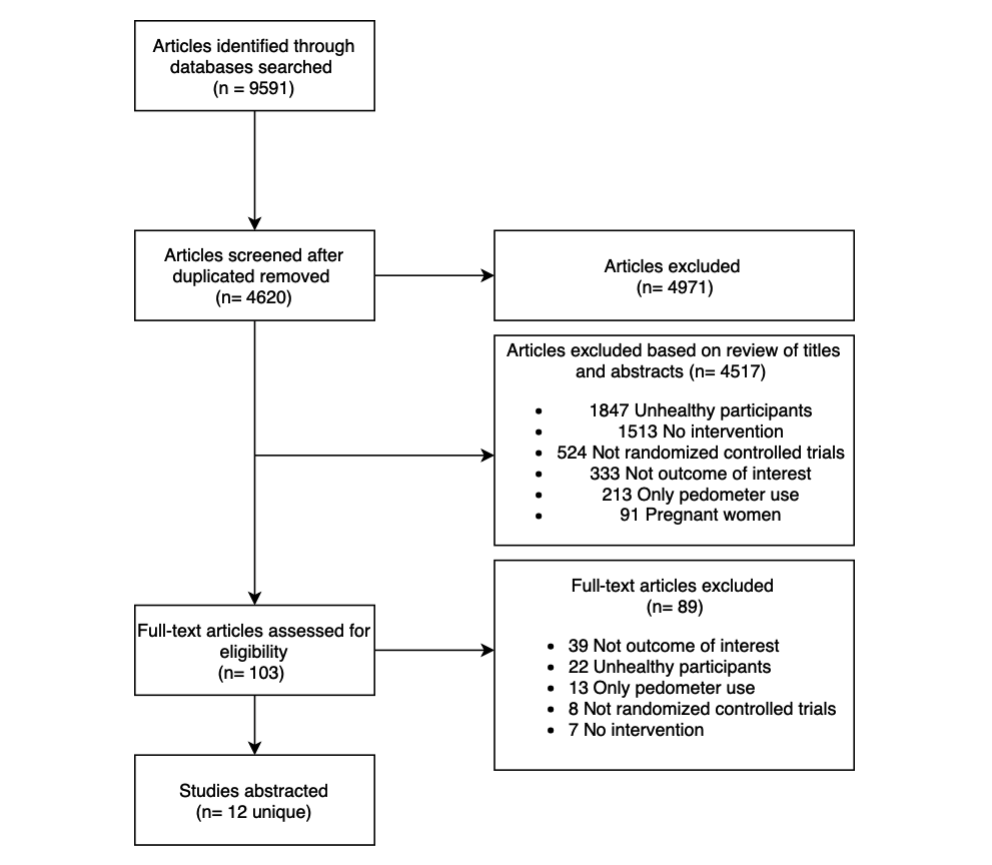
PRISMA Flow Chart.

The primary search identified a total of 9591 studies from MEDLINE (n=1910), CINAHL (n=62), Cochrane Library (n=2871), Web of Science (n=1633), PubMed (n=1280), and Scopus (n=1835), as detailed in Multimedia Appendix 1. There were 4620 unique citations in which 103 full texts were retrieved for further review after applying the Population, Intervention, Comparison, Outcome, and Study type [18] criteria of inclusion and exclusion studies (Multimedia Appendix 2) at the title-and-abstract screening level. A total of 12 unique papers were retained for data abstraction (Figure 1) [19].

### Study Characteristics

Characteristics of the included studies are presented in Table 1, and the intervention and comparators are presented in Table 2. A total of 1693 participants were included from 12 randomized trials. Included studies were published from 2007 to 2017.

Study sample sizes varied from 21 to 471 participants. The participants’ weighted average age was 40.7 years (95% CI 31.1-50.3), and 64.4% of the participants were women. The duration of interventions ranged from 6 to 104 weeks, with a mean intervention duration of 21.4 weeks (95% CI 6.1-36.7).

**Table 1:** Characteristics of participants in the included studies.

Study name, publication year	Sample size	Participant characteristics			Study duration (weeks)
		Health status	Mean age (years)	Proportion	
Ashe, 2016 [20]	25	Healthy participants	40.1	Women: 100%	26
Buis, 2017 [21]	40	Healthy overweight adults	61.5	Women: 66%; white ethnicity: 66%	12
Cadmus-Bertram, 2015 [22]	51	Healthy and overweight participants	60.0	White ethnicity: 92%	16
Godino, 2013 [23]	466	Healthy participants	47.7	Women: 46%; white ethnicity: 96%	8
Hurling, 2007 [24]	77	Healthy participants	40.3	Women: 66%; white ethnicity: 99%	9
Jakicic, 2016 [25]	471	Healthy overweight adults	30.9	Women: 71%; white ethnicity: 77%	104
Martin, 2015 [26]	49	Healthy overweight adults	58.0	Women: 46%; white ethnicity: 79%	5
Melton, 2016 [27]	69	Healthy participants	19.7	Women: 100%; black ethnicity: 100%	6
Poirier, 2007 [28]	264	Healthy participants	39.9	Women: 66%; white ethnicity: 77%	7
Shrestha, 2013 [29]	28	Healthy overweight adults	32.1	Women: 54%	26
Thompson, 2014 [30]	49	Healthy participants	79.1	Women: 91%; white ethnicity: 66%	26
Thorndike, 2014 [31]	104	Healthy participants	29.0	Women: 54%	12

**Table 2:** Characteristics of intervention and comparators of included studies.

Study Name, Year	Intervention Device	Intervention	Comparator
Ashe, 2016 [20]	Fitbit	26 weeks of group-based education, social support, individualized physical activity prescription, given Fitbit	26 weeks: only received health-related information
Buis, 2017 [21]	Jawbone Up24	Received a Jawbone Up24 monitor, a tablet with Jawbone Up app installed, and brief weekly telephone counseling	Waitlist control (did not receive any intervention until after their final assessment where they were provided the intervention in full)
Cadmus-Bertram, 2015 [22]	Fitbit	16 weeks of Web-Based Tracking Group: Fitbit, instructional session, follow-up call at the fourth week	16 weeks of standard pedometer
Godino, 2013 [23]	Combined HR monitor and accelerometer (Actiheart)	8 weeks of wearing of Actiheart with one of three different types of feedback (simple, visual, contextualized)	8 weeks of wearing of Actiheart but with no feedback until the end of the trial
Hurling, 2007 [24]	Wrist-worn accelerometer	9 weeks of wristworn accelerometer, weekly exercise schedule, email reminders, real-time feedback via the internet	9 weeks of wrist-worn accelerometer with no feedback
Jakicic, 2016 [25]	FIT Core; BodyMedia	24 weeks of enhanced intervention: wearable technology, accompanying web interface to monitor diet and physical activity	24 weeks of standard intervention: website for self-monitoring of diet and physical activity
Martin, 2015 [26]	Fitbug Orb	3-arm study Phase 1 (1 week): blinded run-in Phase 2 (2 weeks): unblinded versus blinded tracking Phase 3 (2 weeks): smart texts versus no texts	Blinded participants with no feedback
Melton, 2016 [27]	Jawbone UP	6 weeks of wearing Jawbone UP band and engaging with the application daily with weekly reminders	6 weeks of using MyFitnessPal application
Poirier, 2007 [28]	Variety of activity trackers	2-arm study 6 weeks of walking program, Walkadoo, and wireless activity tracker 1 week of follow-up with wearing of activity tracker for at least 10 hours a day	2-arm study 6 weeks of not wearing activity trackers and maintaining daily activity routine 1 week of follow-up wearing of activity tracker for 10 hours a day
Shrestha, 2013 [29]	Polar FA20 accelerometer	1 time 1.5-hour lifestyle instruction and 26 weeks of continuous accelerometer use and feedback	26 weeks of self-directed exercise and/or US Army mandated physical training
Thompson, 2014 [30]	Fitbit	26 weeks of accelerometer use and feedback, weekly brief telephone counseling sessions focused on accelerometer feedback, 6 in-person brief counseling sessions	26 weeks of accelerometer without feedback
Thorndike, 2014 [31]	Fitbit e3	2-arm study Phase 1: 6 weeks RCT^a^ comparing daily steps displaying feedback about steps and energy consumed Phase 2: 6 weeks non-RCT team steps competition where all participants wore monitors with feedback	2-arm study Phase 1: 6 weeks blinded monitor Phase 2: 6 weeks non-RCT team steps competition where all participants wore monitor with feedback

^a^RCT: randomized controlled trial.

### Impact of Wearable Tracker Use and Physical Activity

**Figure 2:**
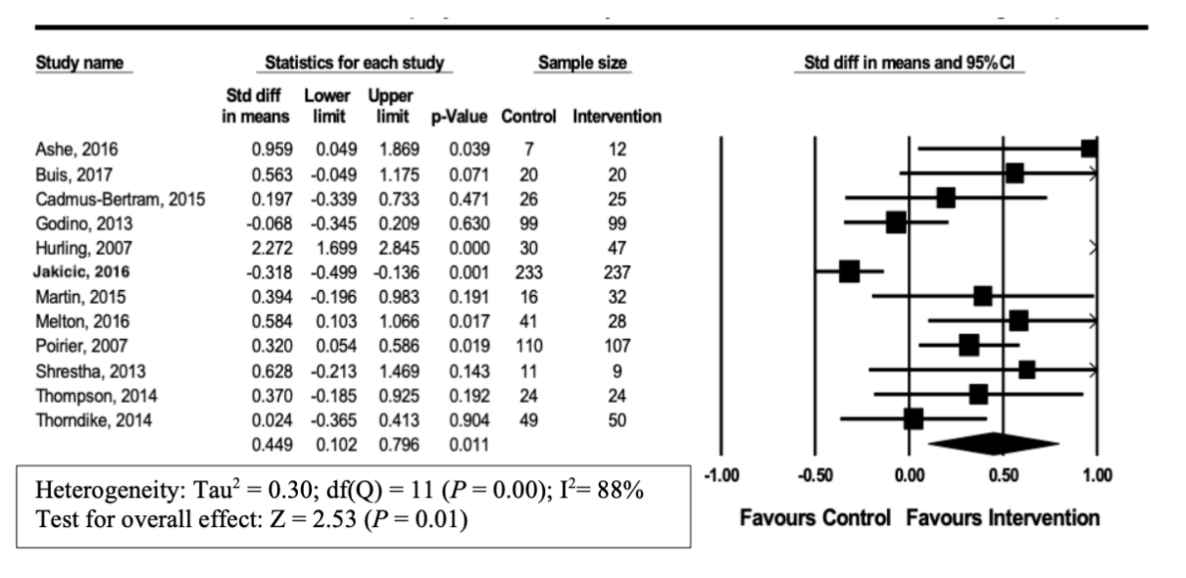
Forest plot of standardized mean difference (95% CI) in the effect of wearable trackers on physical activity.

The primary outcome for this review was the impact of Wearable Tracker Use and Physical Activity. The overall summary estimate from 12 studies [20-31] showed a modest increase in physical activity with the usage of wearable trackers (SMD 0.449, 95% CI 0.10-0.80; *P*=.01). There was significant heterogeneity (I^2^=88%) (Figure 2). Subgroup analyses were performed for studies using steps/day or weight as reported endpoints, and in healthy versus overweight populations to explore mechanisms of heterogeneity.

We performed subgroup analyses and assessed heterogeneity to evaluate the robustness of our results [32].

### Impact of Wearable Tracker Use in Studies With Steps/Day as Primary Outcome Variable

**Figure 3:**
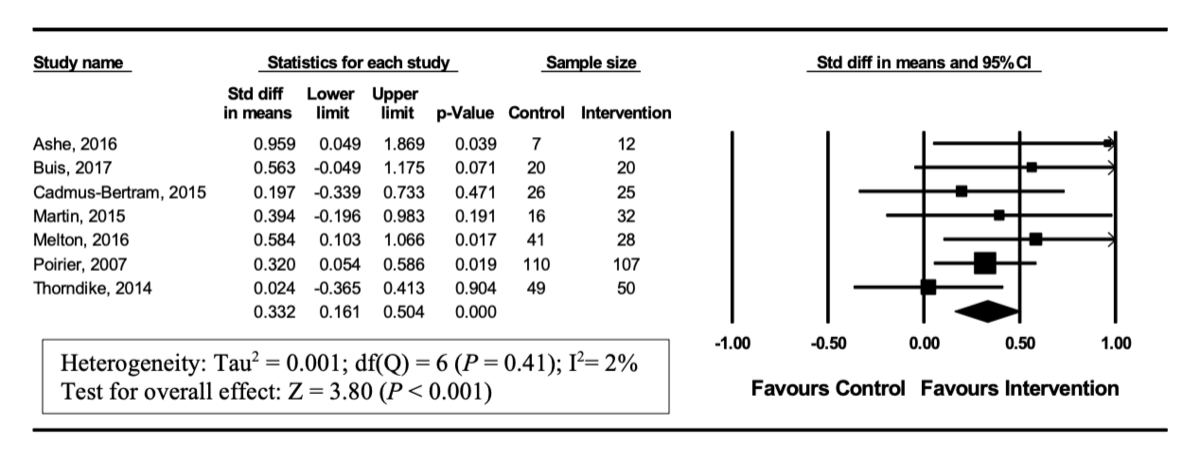
Forest plot of standardized mean difference (95% Cl) in the effect of wearable trackers on steps/day.

A total of 7 [20-22,26-28,31] of 12 studies utilized steps/day as the primary endpoint. In these studies, steps/day were significantly increased by the end intervention (SMD 0.332, 95% CI 0.16-0.50; *P<*.001). Heterogeneity in this analysis was low (I^2^=2%) (Figure 3).

### Impact of Wearable Tracker Use in Studies With Weight Loss as an Outcome

**Figure 4:**
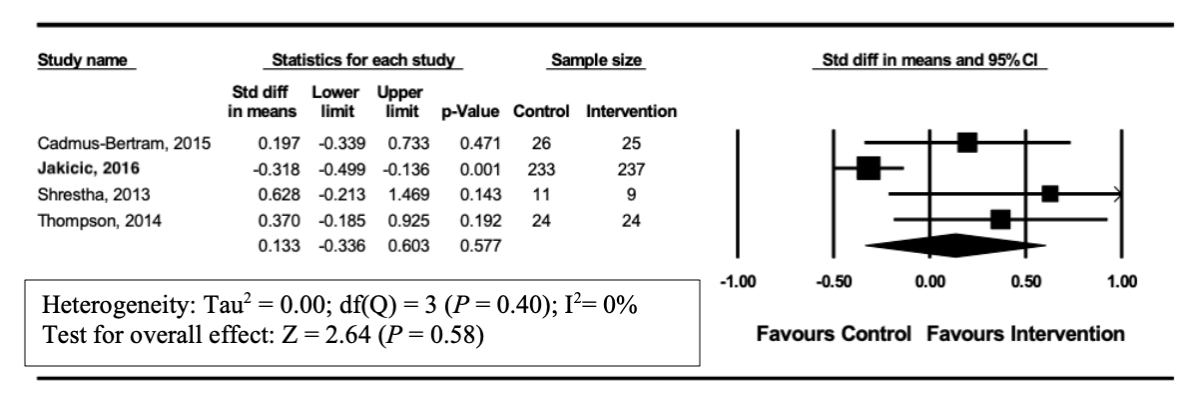
Forest plot of standardized mean difference (95% Cl) in the effect of wearable trackers on weight loss for intervention and control group.

A total of 4 [22,25,29,30] of 12 studies reported weight change. However, no significant effect on weight change was observed (SMD 0.133, 95% CI –0.34 to 0.60, *P*=.58). Heterogeneity in this analysis was low (I^2^=0%) (Figure 4).

### Impact of Wearable Tracker Use in Studies on Overweight Reduction

**Figure 5:**
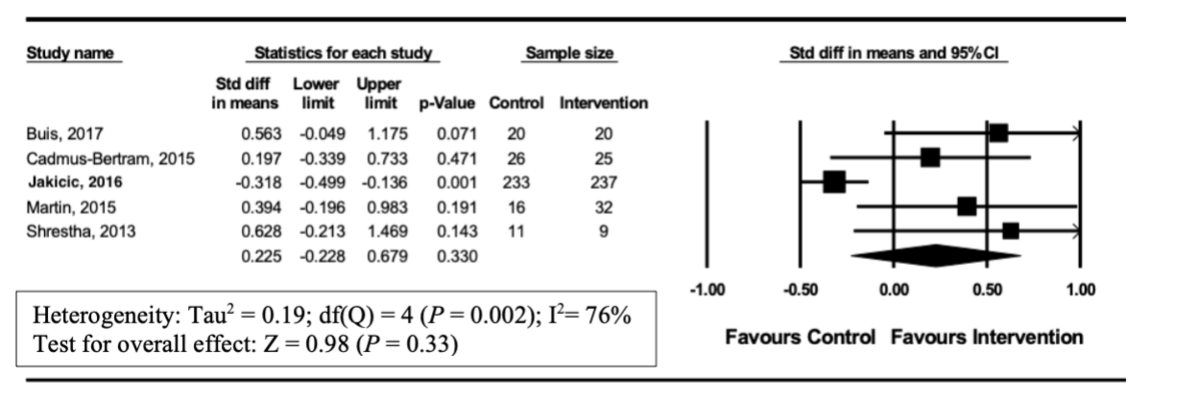
Forest plot of standardized mean difference (95% CI) in the effect of wearable trackers on physical activity in overweight adults.

**Figure 6:**
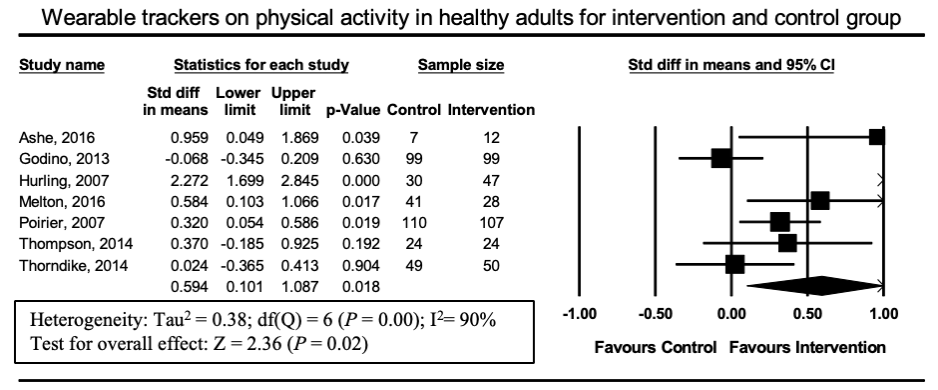
Forest plot of standardized mean difference (95% CI) in the effect of wearable trackers on healthy adults.

A total of 5 [21,22,25,26,29] of 12 studies reported physical activity outcomes in overweight adults. In these studies, no significant increase in physical activity occurred (SMD 0.225, 95% CI –0.23 to 0.68, *P*=.33). Heterogeneity in this analysis was high (I^2^=76%) (Figure 5). Seven [20,23,24,27,28,30,31] out of 12 studies reported physical activity outcomes in healthy adults unselected by weight. In these studies, a significant increase in physical activity was observed (SMD 0.594, 95% CI 0.10-1.09; *P*=.02) Heterogeneity in this analysis was high (I^2^=90%; Figure 6).

### Synthesis of Results

**Figure 7:**
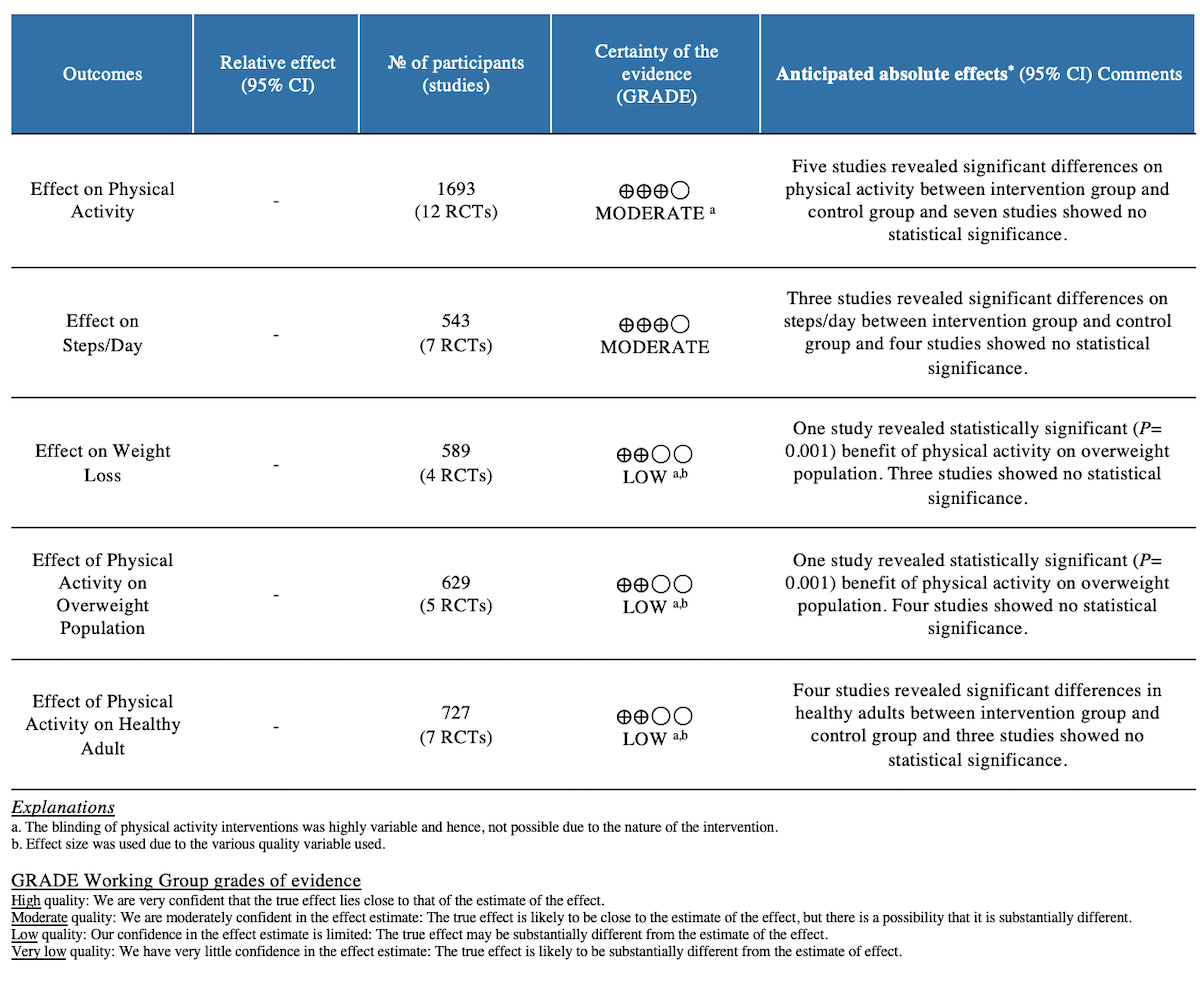
GRADE Working Group grades of evidence summary.

A GRADE summary of results is presented in Figure 7. We assessed outcomes using the Cochrane GRADE approach. Results were downgraded when there was serious risk of bias, inconsistency, indirectness, imprecision, upgrading of a large effect size, or a dose-response gradient, all of which are possible confounding effects. Such confounding effects may create the appearance of an effect when there is none or reduce the appearance of an effect [33]. A GRADE Summary of Evidence table is provided in Multimedia Appendix 4.

### Risk of Bias Across Results

Risk of bias (ROB) is measured using the Cochrane risk of bias tool [13] for randomized controlled trials, and the summary of the evaluation is shown in Table 3. Selection bias was low as randomization was considered high in all of the studies. In 2/12 studies [20,28], methods for allocation concealment were described in insufficient details resulting in high ROB, and one [21] of the studies showed an unclear ROB. For the outcome of physical activity, the blinding of participants and personnel was highly variable as there are challenges to blinding physical activity interventions.

Most of the trials (9/12, 58%) [20-25,27,28,30] did not provide sufficient methodological detail to judge bias not covered within other domains mentioned and were given judged to have an unclear ROB. The remaining studies (25%) provided sufficient details and judged to be low ROB [26,29,31].

Reporting bias was judged to be at low ROB because most of the trials (9/12, 75%) [21-26,29-31] reported details of the measured outcomes that were sufficient.

Detection bias was judged to be low since most of the trials (10/12, 83%) [20-30] provided sufficient information regarding outcome blinding assessment, and the remaining trials (16%) provided insufficient information.

Attrition bias was assessed to be low since all the trials reported the numbers reported to each group. The majority of trials (11/12, 92%) [20,21,23-31] included information on attrition and excisions from the analysis. One trial [22] did not disclose the reason for attrition/exclusion in sufficient detail, resulting in a judgment of high ROB.

Of the 12 studies measuring physical activity as an outcome, only 2 were judged to be of high ROB in terms of performance bias due to lack of blinding, and 1 study showed inadequate information regarding blinding.

Overall ROB was assessed, and the majority of studies (9/12, 75%) [21,23-30] were judged to be at low ROB and the remaining studies [20,22,31] were judged at unclear ROB.

Publication bias was determined by visual inspection of funnel plots comparing physical activity against effect size. There was visual evidence of publication bias with at least two studies falling outside the range of expected precision for their effect size (Multimedia Appendix 3).

**Table 3:** Risk of bias (Cochrane Critical Appraisal Skills Program Tool^a^).

	Q1	Q2	Q3	Q4	Q5	Q6	Q7	Total score/7	Quality Score, %
Ashe, 2016 [20]	L^b^	L	H^c^	U^d^	H	L	L	4.5	64
Buis, 2017 [21]	L	L	U	U	U	L	L	5.5	79
Cadmus-Bertram, 2015 [22]	L	L	L	U	U	U	H	4.5	64
Godino, 2013 [23]	L	L	L	U	L	L	L	6.5	93
Hurling, 2007 [24]	L	L	L	U	H	L	L	5.5	79
Jakicic, 2016 [25]	L	L	L	U	L	L	L	6.5	93
Martin, 2015 [26]	L	L	L	U	H	L	L	5.5	79
Melton, 2016 [27]	L	L	L	L	U	L	L	6.5	93
Poirier, 2007 [28]	L	L	H	U	L	L	L	5.5	79
Shrestha, 2013 [29]	L	H	L	L	L	L	L	6	86
Thorndike, 2014 [31]	L	U	L	L	H	U	L	5	71
Thompson, 2014 [30]	L	H	L	U	L	L	L	6	86
Category Score (%)	100	88	79	45	54	88	100		

^a^Cochrane risk of bias tool. Q1: Were there selection bias (biased allocation to interventions) due to inadequate generation of a randomized sequence? Q2: Were there selective bias (biased allocation to interventions) due to inadequate concealment of allocations prior to assignment? Q3: Were there reporting bias due to selective outcome report? Q4: Were there bias due to problems not covered elsewhere in the table? Q5: Were there performance bias due to knowledge of the allocated interventions by participants and personnel during the study? Q6: Were there detection bias due to knowledge of the allocated interventions by outcome assessors? Q7: Were there attribution bias due to amount, nature, or handling or incomplete outcome data?

^b^L: low risk.

^c^H: high risk.

^d^U: unclear risk.

## Discussion

This meta-analysis examined the effects of wearable trackers on physical activity and is based on 12 randomized controlled trials involving 1693 participants. Overall, wearable tracker usage was associated with improvements in physical activity (SMD 0.594, 95% CI 0.10-1.09; *P*=.018). Interventions that included a consumer-based wearable tracker demonstrated an improvement in physical activity as compared to control groups, especially with daily steps. No clear evidence of benefit was seen from wearable tracker use in the endpoints of weight reduction or physical activity of overweight populations. Indeed, a recent review showed a potential increase in physical activity but no evidence for its effectiveness in weight loss [34].

### Public Health Implications

This data may have both individual and public health implications. Wearable trackers can support continuous health monitoring at both the individual and the population level. Wearable trackers are activity monitoring tools that help to engage patients as advocates in their personalized care and have been proposed to encourage healthy behavior. Benefits are thought to include prevention or reduction of health problems, support of chronic disease self-management, enhanced provider knowledge, reduced number of healthcare visits, and personalized, localized, and on-demand interventions in ways not previously possible [35]. The low cost of delivery and the feasibility of wearable trackers makes them an attractive potential tool to facilitate self-monitoring of physical activity. The data presented in this meta-analysis demonstrates that wearable tracker usage was associated with short-term gains in physical activity [36].

A wide range of wearable trackers was used. Four [20,22,30,31] of 12 studies used Fitbit, two [21,27] used Jawbone Up, and the remaining studies used a variety of other wearable trackers. The relative proportion of commercial wearable trackers in the included studies is similar to global market shares, with Fitbit having the largest market share (20%) and hence applicable to the real world [37]. The Fitbit and Jawbone Up used in this meta-analysis have the same selected measures of “steps, distance, calories, and sleep” and are worn on the wrist [21,22,27,30,31]. A systematic review assessing the validity and reliability of Fitbit and Jawbone found that the validity and inter-device reliability of steps counts was generally high [10].

### Study Limitations

This study has several limitations. First, most studies included in this meta-analysis included small study sizes with short intervention durations and limited follow-up, highlighting the necessity for longer-term studies. The variety of study designs may have been reflected by the statistical heterogeneity of the outcomes. Hence, it might be challenging to generalize the results due to heterogeneity. Future research should explore the long-term effectiveness of wearable trackers in increasing physical activity. Second, statistical estimates of publication bias identified individual, small studies with relatively large effect sizes, which may be a reflection of a file drawer effect. Third, it is difficult to establish the independent contribution of adjunctive interventions (eg, behavioral counseling, interactive health coach, weekly reminders, and text messages) that were often offered alongside wearable tracker usage. The last study was obtained in 2017, since which time there have been changes in market share due to the volatile nature of the industry. The entrance of new brands like Apple, Xiaomi, and Samsung means that an updated review is needed. Next, the skewed demographics to white females would mean that the translation to other demographics might be limited. Lastly, we restricted our search to only full-text published articles in the English language and, thus, may have excluded relevant studies outside our current scope.

### Recommendations for Further Research

The current study examined the utility of wearable activity trackers in healthy adults and found a modest but measurable benefit in the population studied. Important questions for future research could include the identification of appropriate deployment strategies for these novel technologies in cardiac rehabilitation, aged care, and youth. A related question that we could not address was the role of social engagement in modulating the response of participants to wearable tracking devices.

### Recommendations for Clinical Practice

The current study provides qualified support for the use of wearable activity trackers in healthy populations, showing evidence of short-term gains in physical activity, but not weight. As technology advances and these devices improve over time, future studies will be necessary to delineate the optimal use case for these devices.

### Conclusion

In conclusion, this meta-analysis demonstrates the efficacy of wearable trackers in facilitating short-term increases in consumer physical activity. Future studies will be required to determine the durability of the influence of wearable tracker use on consumer physical activity behavior.

## Appendix

Multimedia Appendix 1 Search strategies.

Multimedia Appendix 2 PICOS criteria for inclusion and exclusion of studies.

Multimedia Appendix 3 Funnel plot showing publication bias.

Multimedia Appendix 4 GRADE Summary of Evidence Table.

## Abbreviations

GRADE: Grades of Recommendation, Assessment, Development, and Evaluation
MVPA: moderate-vigorous physical activity
RCT: randomized controlled trial
ROB: risk of bias
SMD: standardized mean difference
