# Supplementary material for: Effectiveness of Wearable Trackers on Physical Activity in Healthy Adults: Systematic Review and Meta-Analysis of Randomized Controlled Trials
Source: JMIR Mhealth Uhealth. 2020 Jul 22;8(7):e15576. doi: 10.2196/15576 (PMC7407266; doi:10.2196/15576)
Supplement: Multimedia Appendix 1 [file mhealth_v8i7e15576_app1.docx]

**Multimedia Appendix 1: Search Strategies**

**Database: MEDLINE**

*Search Date: 01/08/2017*

| Set | Search Terms | Results |
| --- | --- | --- |
| 1 | Telemedicine/ | 16715 |
| 2 | Accelerometry/ | 3014 |
| 3 | Actigraphy | 2611 |
| 4 | Magnetometry | 207 |
| 5 | (accelerometer* or act?graph* or fitbit* or jawbon* or pedometer* or Withings Pulse or misfit* or miband or gps or Global position* system*).tw,kw. | 39010 |
| 6 | (Wear* adj3 ((activi* or fit* or step* or move* or moving or motion*) and (track* or monitor* or count* or technology))).tw,kw. | 1674 |
| 7 | or/1-6 | 58421 |
| 8 | Motor Activity/ | 92849 |
| 9 | Movement/ or exercise/ or muscle stretching exercises/ or physical conditioning, human/ or resistance training/ or running/ or jogging/ or swimming/ or walking/ or warm-up exercise/ or physical exertion/ or physical fitness | 278064 |
| 10 | Physical Fitness/ | 25966 |
| 11 | (move* or step* or activit* or walk* or exercis* or run* or fit*).tw,kw. | 4033058 |
| 12 | or/8-11 | 4140628 |
| 13 | 7 and 12 | 21807 |
| 14 | limit 13 to (English and humans and randomized controlled trial) | 1910 |

**Database: CINAHL**

*Search Date: 01/08/2017*

| Set | Search Terms | Results |
| --- | --- | --- |
| S1 | (MH "Telehealth") OR (MH "Telemedicine") | 7536 |
| S2 | (MH "Accelerometry") | 2190 |
| S3 | (MH "Actigraphy") | 211 |
| S4 | TI (accelerometer* or act?graph* or fitbit* or jawbon* or pedometer* or Withings Pulse or misfit* or miband or gps or Global position* system*) OR AB (accelerometer* or act?graph* or fitbit* or jawbon* or pedometer* or Withings Pulse or misfit* or miband or gps or Global position* system*) | 9403 |
| S5 | TI ((Wear* N3 ((activi* or fit* or step* or move* or moving or motion*)) and (track* or monitor* or count*))) OR AB ((Wear* N3 ((activi* or fit* or step* or move* or moving or motion*)) and (track* or monitor* or count*))) OR (wearable technology) | 207 |
| S6 | S1 OR S2 OR S3 OR S4 OR S5 | 18241 |
| S7 | (MH "Motor Activity") OR (MH "Physical Activity") OR (MH "Human Activities") | 27935 |
| S8 | (MH "Exercise") OR (MH "Abdominal Exercises") OR (MH "Aerobic Exercises") OR (MH "Jumping") OR (MH "Aquatic Exercises") OR (MH "Aerobic Dancing") OR (MH "Running") OR (MH "Jogging") OR (MH "Walking") OR (MH "Anaerobic Exercises") OR (MH "Group Exercise") OR (MH "Back Exercises") OR (MH "Physical Fitness") OR (MH "Physical Performance") OR (MH "Sports+") OR (MH "Aquatic Sports") OR (MH "Cycling") | 89866 |
| S9 | TI (move* or step* or activit* or walk* or exercis* or run* or fit*) OR AB (move* or step* or activit* or walk* or exercis* or run* or fit*) | 326449 |
| S10 | S7 OR S8 OR S9 | 377123 |
| S11 | S6 AND S10 | 5871 |
| S12 | (randomized controlled trials or RCTs OR randomised controlled trials) | 70731 |
| S13 | S11 AND S12 | 62 |

**Database: COCHRANE**

*Search Date: 01/08/2017*

| Set | Search Terms | Results |
| --- | --- | --- |
| #1 | ((move* OR step* OR activit* OR walk* OR exercis* OR run* OR fit*)) | 215,448 |
| #2 | ((accelerometer* OR act?graph* OR fitbit* OR jawbon* OR pedometer* OR "Withings Pulse" OR misfit* OR miband OR gps OR "Global position* system*") OR ((Wear* NEAR/3 (activi* OR fit* OR step* OR move* OR moving OR motion*) NEAR/2 (track* OR monitor* OR count*)))) | 5550 |
| #3 | ((Randomized controlled trial* OR Randomised controlled trials OR RCTs)) | 823,173 |
| #4 | #3 AND #2 AND #1 | 2871 |

**Database: WEB OF SCIENCE**

*Search Date: 01/08/2017*

| Set | Search Terms | Results |
| --- | --- | --- |
| #1 | ((move* OR step* OR activit* OR walk* OR exercis* OR run* OR fit*)) | 6,634,670 |
| #2 | ((accelerometer* OR act?graph* OR fitbit* OR jawbon* OR pedometer* OR "Withings Pulse" OR misfit* OR miband OR gps OR "Global position* system*") OR ((Wear* NEAR/3 (activi* OR fit* OR step* OR move* OR moving OR motion*) NEAR/2 (track* OR monitor* OR count*)))) | 121, 695 |
| #3 | ((Randomized controlled trial* OR Randomised controlled trials OR RCTs)) | 364, 765 |
| #4 | #3 AND #2 AND #1 | 1633 |

**Database: PUBMED**

*Search Date: 01/08/2017*

| Set | Search Terms | Results |
| --- | --- | --- |
| #1 | ((move* OR step* OR activit* OR walk* OR exercis* OR run* OR fit*)) | 4,089,309 |
| #2 | ((accelerometer* or act?graph* or fitbit* or jawbon* or pedometer* or Withings Pulse or misfit* or miband or gps or Global position* system* or (Wear* AND ((activi* or fit* or step* or move* or moving or motion*) AND (track* or monitor* or count*)))) NOT Medline [sb]) | 2423 |
| #3 | #2 AND #1 | 1280 |

**Database: SCOPUS**

*Search Date: 01/08/2017*

| Set | Search Terms | Results |
| --- | --- | --- |
| #1 | ((move* OR step* OR activit* OR walk* OR exercis* OR run* OR fit*)) | 9,861,880 |
| #2 | ((accelerometer* OR act?graph* OR fitbit* OR jawbon* OR pedometer* OR "Withings Pulse" OR misfit* OR miband OR gps OR "Global position* system*") OR ((Wear* NEAR/3 (activi* OR fit* OR step* OR move* OR moving OR motion*) NEAR/2 (track* OR monitor* OR count*)))) | 180,103 |
| #3 | ((Randomized controlled trial* OR Randomised controlled trials OR RCTs)) | 652,931 |
| #4 | #3 AND #2 AND #1 | 1835 |
