# Supplementary material for: Effectiveness of Wearable Trackers on Physical Activity in Healthy Adults: Systematic Review and Meta-Analysis of Randomized Controlled Trials
Source: JMIR Mhealth Uhealth. 2020 Jul 22;8(7):e15576. doi: 10.2196/15576 (PMC7407266; doi:10.2196/15576)
Supplement: Multimedia Appendix 2 [file mhealth_v8i7e15576_app2.docx]

| Study Characteristics | Inclusion Criteria | Exclusion Criteria |
| --- | --- | --- |
| Population | Healthy Adults | Unhealthy adults (cancer, COPD, asthma, knee/hip replacement surgeries) and pregnant women |
| Interventions | Studies will be included if at least one of the groups used wearable trackers that provide objective feedback on physical activity to the wearable, alone or in combination with other interventions to enhance physical activity | Pedometer based (only) studies, non-wearable systems, and systems that do not objectively monitor activity or provide feedback to the wearer |
| Comparators | Wearable trackers and specific brands | Pedometer-based interventions and other active comparator focused on enhancing physical activity |
| Outcomes | Change in physical activity behavior (total steps, total activity, the proportion of participants at activity goal) | Not listed outcomes |
| Setting | Outpatient setting and community settings | Intervention delivered primarily in hospital inpatient setting and studies where monitoring of physical activity is confined to a supervised setting (eg, research center) |
| Study Design | RCTs, n>20 | Not a clinical study (eg, editorial, non-systematic review, letter to the editor, case series), prospective and retrospective observational studies |
| Publication Type | English language only | Non-English articles and abstracts only |
