# Supplementary figures and images for: Effectiveness of Wearable Trackers on Physical Activity in Healthy Adults: Systematic Review and Meta-Analysis of Randomized Controlled Trials
Source: JMIR Mhealth Uhealth. 2020 Jul 22;8(7):e15576. doi: 10.2196/15576 (PMC7407266; doi:10.2196/15576)

**Multimedia Appendix 3: Funnel plot showing publication bias**


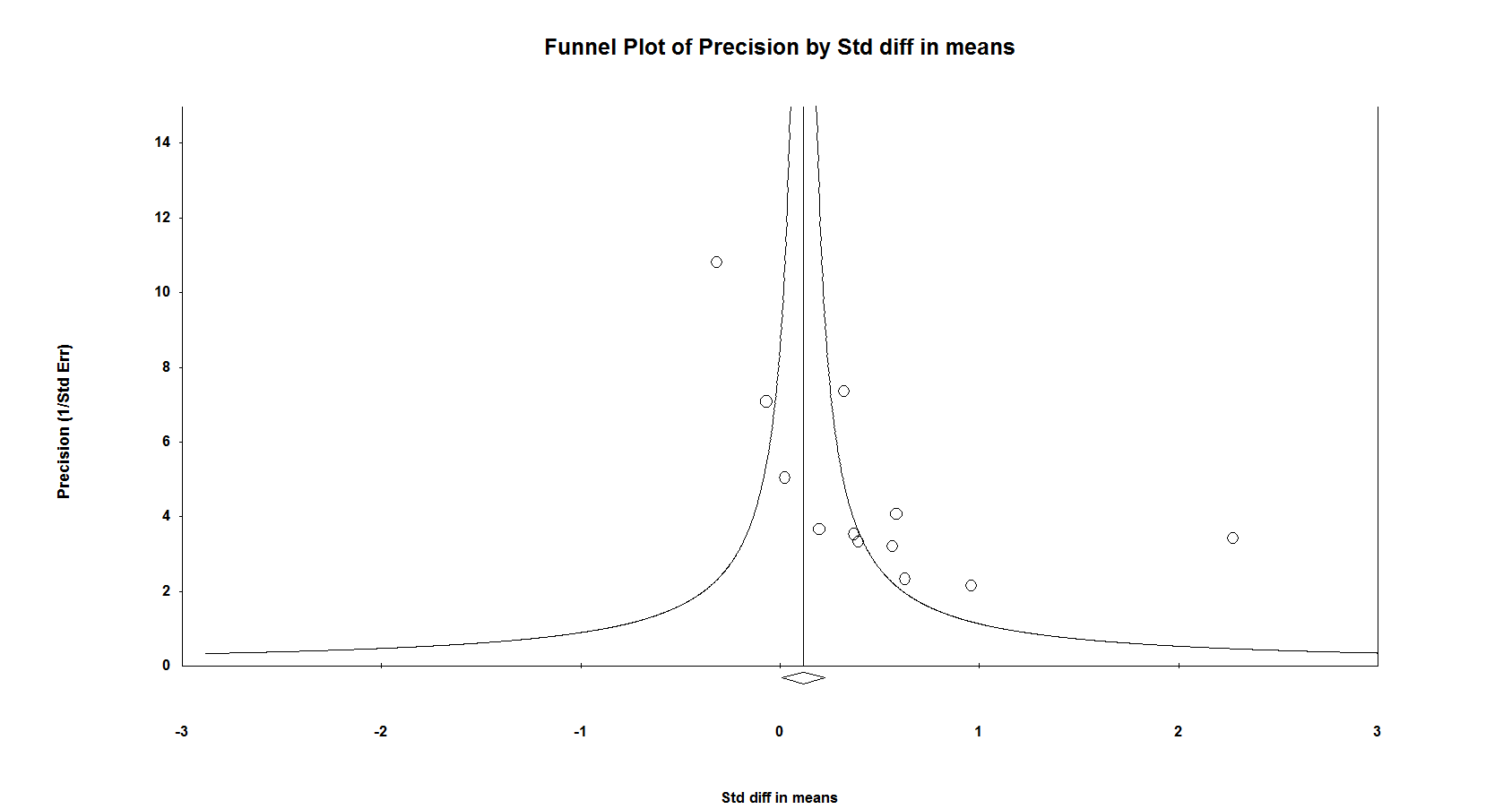

Supplement: Multimedia Appendix 3 [file mhealth_v8i7e15576_app3.docx]
