# Supplementary material for: Effectiveness of Wearable Trackers on Physical Activity in Healthy Adults: Systematic Review and Meta-Analysis of Randomized Controlled Trials
Source: JMIR Mhealth Uhealth. 2020 Jul 22;8(7):e15576. doi: 10.2196/15576 (PMC7407266; doi:10.2196/15576)
Supplement: Multimedia Appendix 4 [file mhealth_v8i7e15576_app4.docx]

| **Certainty assessment** | | | | | | | **№ of patients** | | **Effect** | | **Certainty** | **Importance** |
| --- | --- | --- | --- | --- | --- | --- | --- | --- | --- | --- | --- | --- |
| **№ of studies** | **Study design** | **Risk of bias** | **Inconsistency** | **Indirectness** | **Imprecision** | **Other considerations** | **intervention** | **control group** | **Relative (95% CI)** | **Absolute (95% CI)** |  |  |
| **Effect on Physical Activity** | | | | | | | | | | | | |
| 12 | randomized trials | not serious | serious ^a^ | not serious | not serious | publication bias strongly suspected, all plausible residual confounding would suggest spurious effect, while no effect was observed | 820 | 873 | - | SMD^c^ **0.499 higher** (0.1 higher to 0.8 higher) | ⨁⨁⨁◯ MODERATE | CRITICAL |
| **Effect on Steps/Day** | | | | | | | | | | | | |
| 7 | randomized trials | not serious | not serious | not serious | not serious | publication bias strongly suspected | 274 | 269 | - | SMD **0.332 SD higher** (0.16 higher to 0.5 higher) | ⨁⨁⨁◯ MODERATE | CRITICAL |
| **Effect on Weight Loss** | | | | | | | | | | | | |
| 4 | randomized trials | not serious | serious ^a^ | not serious | serious ^b^ | publication bias strongly suspected, all plausible residual confounding would reduce the demonstrated effect | 295 | 294 | - | MD^d^ **0.133 SD higher** (0.34 lower to 0.6 higher) | ⨁⨁◯◯ LOW | IMPORTANT |
| **Effect of Physical Activity on Overweight Population** | | | | | | | | | | | | |
| 5 | randomized trials | not serious | serious ^a^ | not serious | serious ^b^ | publication bias strongly suspected all plausible residual confounding would reduce the demonstrated effect | 323 | 306 | - | SMD **0.225 SD higher** (0.23 lower to 0.68 higher) | ⨁⨁◯◯ LOW | IMPORTANT |
| **Effect of Physical Activity on Healthy Adult** | | | | | | | | | | | | |
| 7 | randomized trials | not serious | serious ^a^ | not serious | serious ^b^ | publication bias strongly suspected all plausible residual confounding would suggest spurious effect, while no effect was observed | 367 | 360 | - | SMD **0.594 SD higher** (0.1 higher to 1.09 higher) | ⨁⨁◯◯ LOW | IMPORTANT |

^a^ The blinding of physical activity interventions was highly variable and thus not possible due to the nature of the intervention.

^b^ Effect size was used due to the various quality variable used.

^c^ Standardized mean difference

^d^ Mean difference
